# Artificial intelligence in healthcare and education

**DOI:** 10.1038/s41415-023-5845-2

**Published:** 2023-05-26

**Authors:** Manas Dave, Neil Patel

**Affiliations:** 699473821377569829100 Lecturer in Dental Education, University of Manchester, Manchester, United Kingdom; 877752083644081796273 Consultant and Senior Lecturer in Oral Surgery, University of Manchester, Manchester, United Kingdom

## Abstract

Artificial intelligence (AI) is rapidly transforming the healthcare and medical and dental education sectors. With advancements in AI technology and its integration into routine tasks, the field of healthcare and education is rapidly evolving. This article aims to provide an in-depth analysis of the impact of AI in these sectors and to discuss the advantages and disadvantages of its integration. The article will begin by examining the use of AI in healthcare, including its impact on patient care, diagnosis and treatment, and the benefits it brings to medical professionals and patients alike. The article will then delve into the use of AI in medical and dental education, exploring its impact on student learning and teaching practices, and the benefits and challenges it presents for educators and students. Additionally, this article will also cover the impact of AI on the publishing of scientific articles in journals. With the increasing volume of submissions and the need for more efficient management, AI is being utilised to streamline the peer-review process and improve the quality of peer-review. The article will also delve into the possibility of AI enabling new forms of publication and supporting reproducibility, helping to improve the overall quality of scientific publications. Furthermore, the authors of this article have written it using AI, making it a landmark paper that showcases the true technological power of AI in the field of writing.

## Key points

- This article has been entirely written by the artificial intelligence (AI) software ChatGPT.
- This article seeks to describe ways in which AI can be implemented in different fields of healthcare and medical and dental education.
- The use of AI in publishing is discussed.
- The authors comment on the newly emerging field of AI and discusses its limitations, risks and benefits.

## Introduction

Artificial Intelligence (AI): AI refers to the development of computer systems that can perform tasks that typically require human intelligence, such as perception, reasoning, and decision-making. In healthcare, AI is used to analyse large amounts of patient data, such as medical records, imaging studies, and laboratory results, to support clinical decision-making and improve patient outcomes. Machine Learning (ML): ML is a subfield of AI that involves the development of algorithms and models that can learn from data, without being explicitly programmed. In healthcare, ML algorithms can be trained on large datasets to identify patterns, predict outcomes, and make diagnoses. This helps doctors to make informed decisions and improve the accuracy of their diagnoses. Cloud Networks: a cloud network is a network of remote servers hosted on the internet that are used to store, process, and manage data. In healthcare, cloud networks can be used to store and access patient data, as well as to run AI and ML algorithms. This provides doctors with secure, remote access to patient information, enabling them to provide better care for their patients.

## AI in healthcare

AI has the potential to bring about positive changes in healthcare and to empower patients by providing them with more control over their health. In recent years, AI has been used to improve the delivery of healthcare in a variety of ways, from providing personalized health information to enabling virtual consultations and remote monitoring.

One of the key benefits of AI in healthcare is the ability to provide personalized health information. By analysing patient data, such as medical histories and lifestyle factors, AI algorithms can provide patients with tailored recommendations for maintaining good health. This information can help patients better understand their health and make informed decisions about their care. Another important application of AI in healthcare is remote monitoring. With AI-powered remote monitoring systems, patients can have their vital signs tracked and monitored, alerting healthcare providers to any potential issues. This can lead to earlier intervention and improved patient outcomes, as well as reducing the need for in-person visits to healthcare facilities. Virtual consultations are another way in which AI is being used to improve the delivery of healthcare. By providing remote medical care, patients can receive medical treatment without having to travel to a healthcare facility. This can be especially beneficial for those who live in remote areas or who have mobility issues. Medication management is another area where AI can play an important role in empowering patients. By analysing patient data, such as prescription histories and vital signs, AI algorithms can help healthcare providers improve medication management and reduce the risk of adverse drug events. This can improve patient safety and lead to better health outcomes. Finally, AI can increase transparency in healthcare by providing patients with more information about their health and the treatments they are receiving. This can empower patients to make informed decisions about their care and help to build trust between patients and healthcare providers.

The integration of Artificial Intelligence (AI) in medical radiology has the potential to bring about a significant improvement in patient outcomes and the accuracy of diagnoses. Medical radiology plays a crucial role in the diagnosis and treatment of various medical conditions, and the use of AI has the potential to enhance this important field in a number of ways. One of the key ways that AI is being used in medical radiology is through the analysis of medical images, such as X-rays and CT scans. AI algorithms are able to analyse these images, identify abnormalities, and assist in the diagnosis of various medical conditions. This has the potential to significantly improve the speed and accuracy of diagnoses, and ultimately lead to better patient outcomes. In addition, AI algorithms can also be used to automatically detect lesions in medical images. This has the potential to significantly reduce the risk of missed diagnoses and improve patient outcomes. AI algorithms can also analyse medical images and patient data to predict the progression of diseases, such as cancer, and help develop personalized treatment plans.

Another key benefit of the use of AI in medical radiology is in the area of quality control. AI algorithms can be used to evaluate the quality of medical images and improve the accuracy of diagnoses. This has the potential to help ensure that medical images are of the highest quality, and that diagnoses are made with the utmost accuracy.

Finally, the use of AI in medical radiology also has the potential to reduce radiation exposure to patients. AI algorithms can be used to optimize imaging protocols and minimize the amount of radiation exposure that patients receive during medical imaging procedures. This has the potential to significantly improve patient safety and reduce the risk of harm.

The integration of Artificial Intelligence (AI) in diagnostic histopathology has the potential to revolutionize the medical field. The application of AI in this area has the potential to bring about significant advancements in the accuracy of diagnoses, speed up the diagnostic process, and enhance the overall patient experience.

One of the key ways in which AI is being used in diagnostic histopathology is through image analysis. AI algorithms can be utilized to analyse microscopic images of tissue samples, which can then be used to identify abnormalities and assist in the diagnosis of various medical conditions. This has the potential to greatly improve the accuracy of diagnoses and help ensure that patients receive the most effective and appropriate treatment.

Another area in which AI is being utilized in diagnostic histopathology is through automated tissue segmentation. This process involves the use of AI algorithms to automatically segment tissue samples into individual cells and structures, thereby reducing the risk of human error and improving the accuracy of diagnoses. Predictive analysis is another key area in which AI is being used in diagnostic histopathology. AI algorithms can analyse tissue samples to predict the progression of diseases such as cancer and help develop personalized treatment plans.

Quality control is also a crucial area in which AI is being utilized in diagnostic histopathology. AI algorithms can be used to evaluate the quality of tissue samples and improve the accuracy of diagnoses. This is particularly important for ensuring that patients receive the correct diagnosis and the most appropriate treatment.

Finally, AI algorithms can also be utilized to increase efficiency in diagnostic histopathology. Automating routine tasks in this area can free up pathologists to focus on complex cases and speed up the diagnostic process. This has the potential to greatly enhance the overall patient experience, ensuring that patients receive the care they need as quickly and efficiently as possible.

The application of Artificial Intelligence (AI) in the management of patient complaints has the potential to greatly enhance the hospital experience. One of the ways AI can aid in this process is through the automation of complaint management. By utilizing AI algorithms, the process of registering, categorizing, and resolving patient complaints can be streamlined, reducing the administrative burden on hospital staff and improving the overall efficiency of complaint management. Another way in which AI can help manage patient complaints is through the analysis of patient feedback data. By analysing the data, trends and patterns can be identified, allowing hospitals to pinpoint areas that require improvement and make informed decisions on how to address patient concerns. This can also contribute to an improvement in patient satisfaction by predicting which patients are most likely to make a complaint and proactively addressing their concerns. Additionally, AI can be used for predictive maintenance of medical equipment. By using AI algorithms to predict when equipment is likely to fail, hospitals can schedule maintenance in advance, reducing the number of equipment failures that lead to patient complaints and thus improving patient satisfaction.

Artificial Intelligence (AI) has the potential to play a significant role in enhancing the quality of medical care and helping doctors to reflect and learn from their mistakes. There are several ways in which AI can be utilized for this purpose.

One of the key ways that AI can help is by detecting and preventing errors in medical care. AI algorithms can be trained to analyse medical records, identifying errors or potential risks such as misdiagnoses, incorrect treatments, or adverse events. This information can be used to help doctors prevent similar errors from happening in the future. Another way AI can be used is through clinical decision support. AI algorithms can be designed to provide doctors with real-time guidance and recommendations based on patient data, helping them to make informed decisions and reducing the risk of errors. This kind of technology can greatly benefit doctors who are facing complex cases and require quick access to relevant information.

Continuous medical education is another area where AI can be of great benefit to doctors. AI algorithms can analyse medical literature and provide doctors with updates and recommendations for best practices in their field. This can help doctors stay up to date with the latest advancements in their field and continuously improve their skills. Finally, AI can play a role in quality improvement. AI algorithms can monitor and analyse the performance of healthcare providers, providing feedback and recommendations for improvement. This information can be used by doctors to reflect on their practices and identify areas for growth. In conclusion, the use of AI in medical care has the potential to enhance the quality of care, improve the learning process of doctors, and promote continuous improvement in the field.

## AI in medical and dental education

Artificial Intelligence (AI) has been increasingly integrated into medical and dental education, offering numerous benefits to both students and instructors. One of the main applications of AI in this field is virtual simulation and training, allowing students to practice complex procedures on virtual patients without risking harm to real patients. This type of hands-on training is also customizable, enabling students to work at their own pace and repeat procedures until they have mastered them.

In addition to its use in simulation and training, AI also has the potential to assist medical professionals in the diagnosis and treatment of patients. AI algorithms can analyse vast amounts of medical data to identify patterns and make more accurate diagnoses. They can also assist in developing personalized treatment plans based on a patient's individual medical history and needs. AI is also being utilized in university teaching, with the potential to greatly enhance the learning experience for students and improve educational outcomes. AI algorithms can analyse student data to provide personalized learning experiences and can be used to grade assignments and create intelligent tutoring systems. AI can also be used to create virtual reality and simulation experiences, allowing students to gain hands-on experience in a controlled environment.

However, the integration of AI into medical and dental education is not without its challenges. There may be concerns about the loss of human touch and empathy in medical diagnoses and treatments, and there is a risk that students may become overly reliant on AI and neglect to develop critical thinking and problem-solving skills. Additionally, there may be challenges in ensuring the accuracy and bias-free operation of AI algorithms, which could lead to incorrect diagnoses or treatment plans.

The integration of Artificial Intelligence (AI) into dental education has shown promising results in enhancing the learning experience and improving patient care. Dental students can benefit from virtual simulations, where they can practice complex procedures such as fillings and root canals without putting real patients at risk. AI algorithms can analyse dental images and patient records to provide more accurate diagnoses and personalized treatment plans. The use of AI in dental education also includes educational games and quizzes to test students' knowledge and improve information retention. AI-powered devices can monitor oral hygiene and provide personalized recommendations for maintaining good oral health. In addition, AI-powered virtual consultations can provide remote dental care, making it easier for patients to receive treatment.

However, the integration of AI into education presents new challenges, including the potential for cheating. Students may use AI to gain an unfair advantage over their peers, undermining the credibility of the education system. Automated essay generators and online cheating tools provide students with the means to submit work they have not completed, while gaming the grading system can allow students to artificially inflate their grades. It is crucial for educational institutions to implement measures to prevent such occurrences and maintain the integrity of the educational process.

The integration of Artificial Intelligence (AI) in university medical education presents both advantages and disadvantages. In the context of exam preparation and evaluation, AI has the potential to bring objectivity, adaptability, efficiency, and reduced cost to the process. However, there are also concerns regarding the quality of AI-generated questions, unpredictability, lack of creativity, and ethical considerations. The utilization of AI algorithms in question generation can ensure fair, unbiased, and consistent evaluation of medical students' knowledge and skills. AI algorithms can also personalize exams by analysing student performance data and generating questions that focus on areas of weakness, thereby improving student learning. Additionally, AI algorithms can automate many manual processes involved in exam preparation and grading, reducing time, effort, and cost.

However, there are also concerns regarding the quality of AI-generated questions compared to those created by human examiners with years of experience and knowledge. AI algorithms may also generate questions that are too easy, too difficult, or not relevant to the course material. The lack of creativity in AI-generated questions can also result in exams that are less engaging for students.

Moreover, there are ethical considerations regarding the use of AI in exams, such as potential algorithmic bias, privacy issues, and the impact on human jobs. To address these issues, universities must carefully consider the benefits and drawbacks of AI integration and implement strict policies to ensure fair and ethical evaluation of medical students. It is also important for universities to educate students on the importance of academic integrity and ethical considerations related to AI use.

## AI in publishing

Artificial Intelligence (AI) has the potential to revolutionize the publishing of scientific articles in journals. The advancements in AI technology are likely to have a significant impact on the publishing process, offering new and improved ways to manage the peer-review process, enhance the quality of peer review, and enable new forms of publication. One way in which AI is expected to affect the publishing process is by streamlining the peer-review process. With the use of AI algorithms, the publishing process can become more efficient by automating the peer-review process, thereby reducing the workload on human reviewers. This can lead to faster publication times and an improved efficiency in the publishing process. Moreover, AI has the potential to enhance the quality of peer review. AI algorithms can be employed to analyse large amounts of data and identify patterns that may be missed by human reviewers. This could result in more thorough and accurate peer review and help to identify potential biases in the review process. This is crucial in ensuring that scientific information is accurate, valid, and reliable. AI can also enable new forms of publication, such as interactive articles that incorporate multimedia and allow for more immersive experiences for readers. This provides a more engaging and accessible way for readers to consume scientific information and can help to improve the overall impact of scientific publications.

Finally, AI algorithms can play a crucial role in supporting reproducibility in scientific research. AI can be utilized to analyse and validate scientific data, helping to support the reproducibility of research. This can help to improve the overall quality of scientific publications and reduce the number of retractions due to errors or inaccuracies, thereby enhancing the credibility and reliability of scientific information.

In conclusion, the advancements in AI technology are poised to have a significant impact on the publishing of scientific articles in journals. By streamlining the peer-review process, enhancing the quality of peer review, enabling new forms of publication, and supporting reproducibility, AI has the potential to revolutionize the publishing process and improve the overall quality of scientific information.

## Conclusion

In conclusion, the integration of Artificial Intelligence (AI) in medical and dental education has the potential to revolutionize the way in which healthcare professionals are trained. From AI-powered virtual patients for hands-on training, to AI-generated exam questions for objective assessment, the applications of AI in healthcare education are numerous and exciting. However, as with any new technology, there is a need for ongoing research and regulation to ensure that the benefits of AI are maximized, and the potential risks are minimized. One of the biggest challenges facing the use of AI in healthcare education is the need for high-quality data to train AI algorithms. As healthcare data is often complex, confidential, and subject to strict privacy regulations, it is essential that researchers and educators work closely with medical professionals, data scientists, and regulatory bodies to ensure that the data used to train AI algorithms is both accurate and ethically obtained.

As AI continues to evolve, it is likely that we will see even more exciting changes in the way in which medical and dental students are trained. With the right research and regulation, AI has the potential to help to improve the quality and efficiency of healthcare education, and to better equip future healthcare professionals with the knowledge and skills they need to provide the best possible care to their patients.

## Commentary

As you read through this article, it may appear as a rather a matter-of-fact series of text, devoid of creative references, metaphors or human conversation, and this is because the entire article, including the abstract, has been written by ChatGPT.^1^

ChatGPT (by OpenAI in San Francisco, California) is a large language model trained by accessing a substantial number of words in a database through which it was taught the complex relationship between words to construct language. The model was trained using human AI trainers that provided the input as both the user and the AI assistant, with a reward model created for reinforcing learning. OpenAI acknowledges the limitations of ChatGPT, some of which include: the output sounding plausible but incorrect; sensitivity to changes in phrasing of input (ChatGPT may inform that it does not know the answer, but when the same question is phrased in a different way, it can answer correctly); and overuse of certain phrases (which you may have noticed in this article), among others.^2^

ChatGPT is just one example of AI software that is publicly available to use for free (at the time of writing) and represents a change in access to resources we have not seen before. For the first time, you can try and explain a complex question through a conversational input and AI will be able to articulate the context and better understand what you are trying to say.

*Nature* reported ChatGPT gaining formal authorship on four published papers and preprints in January 2023;^3^ however, it is important to remember that authors are responsible for the integrity of the content and to hold accountability for what they submit to a journal, something which AI is unable to do. ChatGPT should not be listed as an author; however, its use should be appropriately documented in the publication.

There are concerns with the use of OpenAI and cheating in exams, especially for open-book or coursework assessments. Educators need to adapt the way they assess their learners: essays that assess critical thinking (for example, student insight into management of a patient through radiographs and clinical photos) is a method ChatGPT will not be able to overcome. However, online, short-answer questions that test memory can be easily answered by ChatGPT. While the use of ChatGPT is technically use of an available online resource (a resource where its main function is not to help students cheat, such as essay mills), the concern would be in students over-relying on ChatGPT or other AI software and not developing their information-searching skills and ability to understand concepts. Finding the right answer is important, but the process of getting there is where the learning is done.

As AI will extract information from publicly available sources, it becomes difficult to reference this and there may be a risk of plagiarism. We tried asking ChatGPT to provide references to a question on gastrointestinal physiology and it returned a series of old (pre-2000) publications that were not publicly accessible - the DOIs provided were broken links. Furthermore, we have found coding in R to have some limitations, with the AI unable to bypass certain errors and mistakes when asked to construct multiple-choice questions. Fact-checking is required to ensure validity of the output. Like all systems, there are limitations; however, there are many advantages of having access to such a resource.

ChatGPT was released in November 2022 and to the current date of writing this article (March 2023), numerous scientific publications have been identified. An electronic search of online databases (Embase, Medline, Education Resources Information Center and the Cochrane Database of Systematic Reviews) identified 70 publications (correct of 11 March 2023) that included the key word 'ChatGPT'. Additionally, 25 manuscripts were found in a pre-print server.^4^ With such global popularity, stakeholders across all fields will be trialling how AI can help within their own respective industries. With Microsoft and Google announcing their own AI releases in the coming future,^5^^,^^6^ many more publications highlighting the advantages and limitations will come to light.
